# A20 targets PFKL and glycolysis to inhibit the progression of hepatocellular carcinoma

**DOI:** 10.1038/s41419-020-2278-6

**Published:** 2020-02-03

**Authors:** Yilu Feng, Ye Zhang, Yi Cai, Ruijie Liu, Miaolong Lu, Tangzhiming Li, Ying Fu, Ming Guo, Huichao Huang, Yifu Ou, Yongheng Chen

**Affiliations:** 1 Department of Oncology, NHC Key Laboratory of Cancer Proteomics, XiangYa Hospital, Central South University, Changsha, 410008 People’s Republic of China; 2 Department of Urology, XiangYa Hospital, Central South University, Changsha, 410008 People’s Republic of China; 3 Department of Pathology, XiangYa Hospital, Central South University, Changsha, 410008 People’s Republic of China; 4 Department of Cardiology, Shenzhen People’s Hospital, Second Clinical Medical College of Jinan University, First Affiliated Hospital of Southern University of Science and Technology, 1017 Dongmen North Road, Shenzhen, Guangdong China; 5 Department of Neurosurgery, The Third Xiangya Hospital, Central South University, Changsha, 410008 People’s Republic of China

**Keywords:** Biochemistry, Cancer metabolism

## Abstract

Abnormal expression of the E3 ubiquitin ligase A20 has been found in some malignant cancers, including hepatocellular carcinoma (HCC). Here, we discovered that A20 is an E3 ubiquitin ligase for phosphofructokinase, liver type (PFKL) in HCC A20 interacts with PFKL and promotes its degradation, therefore inhibiting glycolysis in HCC cell lines. Downregulation of A20 in HCC cells promotes proliferation, migration, and glycolysis, all of which can be inhibited by targeting PFKL with RNA interference. Importantly, A20 is downregulated in advanced HCC tissues and inversely correlated with PFKL expression. Thus, our findings establish A20 as a critical regulator of glycolysis and reveal a novel mechanism for A20 in tumor suppression and PFKL regulation. Given that an increased level of glycolysis is linked with HCC, this study also identifies potential therapeutic targets for HCC treatment.

## Introduction

Ubiquitination-proteasome mediated degradation is a common mechanism by which cells renew their intracellular proteins and maintain protein homeostasis. This process is carried out by three enzymes ubiquitin activating enzyme (E1), ubiquitin-conjugase (E2), and ubiquitin ligase (E3), in which the E3 ubiquitin ligases are responsible for targeting specific substrates (proteins) for ubiquitin-mediated degradation^1,2^. However, in cancer cells, the stability and the balance between oncoproteins and tumor suppressor proteins are disturbed in part due to deregulated E3 ubiquitin ligases. This ultimately leads to either stabilization of oncoprotein(s) or increased degradation of tumor suppressor(s), contributing to tumorigenesis and cancer progression.

A key ubiquitin ligase with consistently altered expression during tumorigenesis is A20, also known as Tumor necrosis factor alpha-induced protein 3 (*TNFAIP3*). A20 is a RING finger domain-containing E3 ligase, which represents a hotspot in immunoregulation^3–6^. It’s ubiquitination substrates include key proteins such as TRAF6, RIP1 and ASK1, which regulate diverse cellular responses including cell inflammation, proliferation, and migration^7^. The overexpression of A20 can inhibits NF-κB signaling transduced from TNF receptors, toll-like receptors, nucleotide-binding oligomerization domain containing 2 (*NOD2*) receptors or T cell receptors, suppressing chronic activation of the immune system and contributing to the development of cancer^3^. It has been identified that A20 is a crucial tumor suppressor in various lymphomas, multiple myeloma (MM) and colorectal carcinoma^8–10^. In addition, a low expression level of A20 was correlated with poor outcomes in these cancers. In contrast, A20 is highly expressed and responsible for the proliferation of glioblastomas, bladder cancer, breast cancer, and gastric cancer^11–14^. Therefore, the role of A20 in different malignances indicate that its biological function is tissue-dependent and has both putative oncoprotein and tumor suppressor functions. Until now, the specific ubiquitination regulatory mechanism of A20 in HCC has remained largely unknown.

Cancer cells exhibit aberrant metabolism characterized by high glycolysis even in the presence of abundant oxygen. This phenomenon, known as the Warburg effect or aerobic glycolysis, facilitates tumor growth with elevated glucose uptake and lactate production^15^. Accumulating evidence suggests that glycolysis is enabled by oncogenic transitions in HCC to facilitate the biosynthesis and metabolism required for cell proliferation and migration^16^. Based on the dramatically increased glucose consumption in cancer cells, targeting enzymes involved in the glycolytic pathway may offer a therapeutic window to modulate HCC glucose metabolism and suppress cancer progression. The glycolytic pathway contains three rate-limiting enzymes, hexokinase (HK), phosphofructokinase (PFK), and pyruvate kinase (PK). Among these enzymes, PFK is the most important rate-limiting enzyme of glycolysis^17^. PFK exists as three isoforms in human: PFKL (liver), PFKM (muscle), and PFKP (platelet)^18^. In addition, PFK has been reported to be regulated by post-translational modifications (PTMs). Several studies have shown that glycosylation and acetylation are crucial for PFK function and stability in the glycolytic pathway, while the mechanisms of other PTMs, such as ubiquitination, have rarely been reported^19^.

In this study, we identified A20 as an important inhibitor of HCC progression through downregulation of glucose metabolism. Mechanistically, A20 decreased the accumulation and stabilization of PFKL by promoting PFKL degradation through UPS, thus reducing glucose consumption and lactate excretion. The level of A20 was negatively correlated with PFKL in HCC cell lines and tissues. These findings establish A20 as a critical regulator of glucose metabolism that suppresses glycolysis to inhibit HCC proliferation and metastasis.

## Materials and methods

### Cell culture and treatment

Huh7 cells were purchased from American Type Culture Collection and HCCLM3 cells were provided by the Cancer Research Institute of Central South University (Changsha, China). All cell lines were used within 3–20 passages of thawing from the original stocks and were tested every 3 months for mycoplasma contamination. The cell lines were maintained for no more than 3 passages between experiments. We used DMEM/ High Glucose medium (Gibco, CT11995500BT) supplemented with 10% fetal bovine serum (Biological Industries, 04-001-1C), 1% penicillin and streptomycin (HyClone, SV30010) to culture Huh7 and LM3 cells as previously described^20^. All cells were maintained in 5% CO_2_ at 37 °C. Proteasomal inhibitor MG132 (Selleckchem, S261916; 10 μM) was added to HCC cells for 6 h. Cells were treated with LPS at different concentrations (0, 9, 18 μg/ml) for 4 h to detect A20 protein changes. Cells were treated with 10 μg/ml CHX for different times (0, 2, 4, 6, 8) to detect PFKL protein changes. The concentration is the final concentration in the culture medium.

### Quantitative real-time PCR assay

Quantitative real-time PCR was performed with the TaqMan PCR mixture (Applied Biosystems) according to standard protocols. The expression of genes was normalized to the expression of the Actin gene. The primers used were: PFKL (F: 5′-CTACGAGGGCTATGAGGGC-3′; R: 5′-GATGACGCACAGGTTGGTGA-3′); A20 (F: 5′-CCGCAAAGTTGGATGAAGCT-3′; R: 5′-TCCATGAGAGAAAGCTGGGG-3′); and β-actin (F: 5′-CATGTACGTTGCTATCCAGGC-3′; R: 5′-CTCCTTAATGTCACGCACGAT-3′).

### Cell migration assay

We suspended 5 × 10^5^ cells with 200 μL serum-free medium and loaded in Transwell chambers (8 μM pore size, Costa). Medium containing 10% FBS was placed in the well below the insert. After incubation for 48 h, cells that migrated through the membrane were fixed in methanol and stained by crystal violet. The number of migrated cells was counted. The photographs were taken from five random fields via microscopy (Olympus, Shinjuku, Tokyo, Japan). Quantitative analysis of the cell migration was performed by Image J. This experiment was repeated three times.

### Cell viability and colony formation assays

A total of 2 × 10^4^ cells were seeded into a well of a six-well plate. After transfection with indicated plasmids, cell numbers were counted every day over 5-day period. Following genetic or chemical perturbation, Huh7 and LM3 cells were collected as a single cell suspension in medium and 2 × 10^3^ cells were seeded into 6-well plates and cultured in 5% CO_2_ at 37 °C for 10 days. Colonies were washed with 1 × PBS for three times. Fixed with 4% paraformaldehyde for 15 min and stained for 15 min with 0.1% crystal violet. These experiments were repeated three times.

### Immunoprecipitation and western blotting

Western blotting was performed as previously mentioned^21,22^. Cells were lysed in 0.1–0.5% Nonidet P40 buffer (Biosharp) (150 mM NaCl, 50 mM Tris-HCl, pH 7.5) containing inhibitors (1 mM phenylmethylsulphonyl fluoride, 1 mg/ml of aprotinin, 1 mg/ml of leupeptin, 1 mg/ml of pepstatin, 1 mM Na3VO4, 1 mM NaF, all at their final concentrations). Debris were removed by centrifuging at 4 °C,12,000 rpm for 15 min. Then, cell lysates were incubated with anti-A20 antibody and protein G agarose (ROCHE,11719416001) at 4 °C. Next, beads were boiled at 100 °C for 15 min and centrifuged at 4 °C for 1 min before loading on 10% SDS-PAGE gels and transferring onto nitrocellulose membranes (Millipore) for western blotting analysis. The primary antibodies to A20 (Cell Signaling Technology, D13H3; with 1:1000 working dilution), A20 (Proteintech, 66695-1-lg; with 1:1000 working dilution), PFKL (Abcam, 18164; with 1:1000 working dilution), Ubiquitin (Cell Signaling Technology, 3936T; with 1:1000 working dilution); ACTB/β-actin (Proteintech, 20538-I-AP; with 1:1000 working dilution), HA (Santa Cruz, sc-57592, with 1:1000 working dilution), and Flag (Sigma F7425; with 1:1000 working dilution) were commercially obtained.

### LC-MS/MS analysis

To identify potential A20-binding proteins, Huh7 cells transduced with pcDNA3-A20 and Huh7 cells transduced with the empty vector were treated with MG132 (10 μM) for 8 h before being collected for assays. A20 was pulled down by IP using anti-A20 antibody and protein G agarose (ROCHE, 11719416001) at 4 °C. LC-MS/MS analysis was performed at Shanghai Applied Protein Technology.

### Glutathione S-transferase precipitation assays

A direct interaction between A20 and PFKL was determined using GST precipitation assays. Briefly, Rosetta (DE3) *Escherichia coli* cells were transformed with the pGEX-6P-1-GST vector or pGEX-6P-1-GST-PFKL, and then, expression was induced using 0.5 mM IPTG at 16 °C for 16 h. The *E. coli* were lysed, and the extracts were incubated with glutathione–Sepharose 4B beads (17075601; GE Healthcare Biosciences AB) at 4 °C for 1 h. The beads were then incubated with purified GFP-tagged A20, which were prepared through IP, for an additional 4 h. Proteins that had interacted were eluted in elution buffer (50 mM Tris-HCl pH 8.0 and 20 mM reduced glutathione) and were subjected to immunoblotting using anti-GFP antibody. Extracts from *E. coli* expressing only a GST tag were used as the negative control.

### Ubiquitin ladder assay

An ubiquitin ladder assay was performed as previously described^23^. 36 h after transfection, cells were collected and lysed in 1% SDS buffer (50 mM Tris-HCl (pH 7.5), 0.5 mM EDTA, 1 mM dithiothreitol) with protease inhibitors (Bimake, b14001) and boiled for 10 min. Before immunoprecipitation, lysates were diluted ten-fold with 0.3% Nonidet P40 buffer. Ubiquitination was determined by western blotting.

### siRNA and shRNA

Downregulation of *PFKL* was performed by RNA interference. Synthetic siRNA oligonucleotides were obtained commercially from Beijing Tsingke Biotech Co., Beijing, China. Sequences of effective sequences were as follows:

Sense: 5′-GCA UCG UCA UGU GUG UCA UTT-3′

Antisense: 5′-AUG ACA CAC AUG ACG AUG CTT-3′

Cells were transfected with lipo2000 (Invitrogen, 11668-027) as described in the standard protocol. The knockdown efficiency was verified by western blotting. The expression plasmid for sh*A20* was made in a pMKO.1-puro vector. The sequences were:

#1 sense: 5′-GCACCGATACACACTGGAAAT-3′

antisense: 5′-ATTTCCAGTGTGTATCGGTGC-3′

#2 sense: 5′-CACTGGAAGAAATACACATAT-3′

antisense: 5′-ATATGTGTATTTCTTCCAGTG-3′

Cells were transfected with Polyethylenimine Linear (Polysciences, 23996-1) as described in the standard protocol.

### Glucose uptake and lactate production

Cells were transfected with pMKO-sh*A20*, pCNDA3-A20, pBABE-PFKL plasmids, and si*PFKL*, respectively, and seeded into 96-well plates. Twenty-four hours after transfection, cellular glucose uptake was measured according to the manufacturer’s instructions (Leadman GLU, China). To measure lactate production, cells were seeded into 6-well plates and transfection was performed as previously described. Lactate production was analyzed using a Lactate Colorimetric Assay Kit (Leadman LAC, China).

### Extracellular acidification rate (ECAR)

ECAR was analyzed on a XF96 Extracellular Flux Analyzer (Seahorse Bioscience) as previously described^24,25^. Cells were plated in non-buffered DMEM media with 10 mM glucose. Measurements were obtained under basal conditions and after the addition of 100 μM oligomycin and 500 mM 2-DG.

### Immunofluorescence staining

Huh7 cells were treated with LPS for 4 h. Thereafter, cells were then fixed in 4% paraformaldehyde (Wuhan Goodbio technology, G1101), permeabilized with 0.2% Triton X-100 (Sigma-Aldrich, T8787), blocked by 5% bovine serum albumin (Amresco, 0332) in phosphate-buffered saline (Sigma-Aldrich, P5368) and then incubated with the indicated primary antibodies: A20 (Proteintech, 66695-1-lg; with 1:1000 working dilution) and PFKL (Abcam, 18164; with 1:1000 working dilution). Detection was performed with corresponding fluorescent-conjugated secondary antibodies (Proteintech, SA00013-2, SA00006-3). Confocal fluorescence images were obtained with a confocal microscope (Zeiss LSM 880 +airy scan). Relative colocalization was calculated with ImageJ software. Mean values were calculated from the individual distributions from 10 cells per condition.

### BiFc Analysis in Huh7 cells

Huh7 cells were maintained in DMEM/ High Glucose medium supplemented with 10% fetal bovine serum, 1% penicillin and streptomycin at 37 °C in a humidified 5% CO_2_ atmosphere. 24 h before transfection, 10^5^ cells were plated on a glass-bottom cell culture dish. Transfections were carried out using Polyethylenimine (PEI), Liner (Polysciences, 23900-1), with a total amount of 4 µg of PEI: 1 µg of the VC155-A20 and 1 µg of VN173-PFKL plasmids. Cells were fixed 24 h after transfection, and coverslips were mounted on glass slides with Vectashiel + Dapi (Vector) and observed under a confocal microscope. For control experiments, fixed cells were subjected to immunohistochemistry using a rabbit polyclonal anti-GFP (Invitrogen, A11122) diluted 1:200 as the primary antibody, and a goat anti-rabbit IgG-AF555 (Molecular Probes 4413) diluted 1:750 as the secondary antibody.

### Proximity ligation assay

In-cell interaction was performed using the Duolink® in situ assay following the manufacturer’s instructions. Briefly, cells were blocked and incubated with anti-A20 (mouse) and anti-PFKL (rabbit) antibodies for 2 h at room temperature, followed by an incubation with anti-rabbit plus and anti-mouse minus PLA® probes (DUO92101-1KT) for 60 min at 37 °C. Ligase was added and incubated for 30 min at 37 °C. The fluorescence signal was then amplified by the addition of polymerase for 100 min at 37 °C. The in-cell complexes were visualized with a confocal microscope (Zeiss LSM 880 + airy scan).

### Xenograft analysis

10 nude mice (nu/nu, 6- to 8-week-old males) were randomly divided into two groups, then injected subcutaneously with 5 × 10^6^ A20 overexpressing or empty vector counterpart stable Huh7 cells. The tumor diameters were measured every 7 days. Approximately 28 days after injection, the tumors were dissected and analyzed. The investigators were blinded to the group allocation during the experiment and when assessing the outcome. The procedures related to animal subjects were approved by the Ethical Committee of the school of Xiangya Hospital of Central South University.

### Liver tumor samples and immunohistochemistry

A total of 10 patients who underwent curative resection for HCC at authors’ institute between 2018 and 2019, were enrolled in the present study. None of them received any preoperative adjuvant treatment. Human liver cancer tissues and their adjacent tissues were obtained with patient informed consent from Xiangya Hospital of Central South University. All procedures were performed under the permission of the Ethical Committee of Xiangya Hospital of Central South University. Immunohistochemistry (IHC) was performed as previously described^26,27^. To quantify the positive staining IHC results, five random areas in each tissue sample were microscopically examined and analyzed by an experienced pathologist. The average staining score was calculated by dividing the positive areas with the total areas. Data obtained were expressed as the mean values ± SD.

### Statistical analysis

A two-tailed Student’s *t*-test was performed and expressed as a *P*-value. GraphPad Prism 7 (for Windows) and Excel were used to perform the statistical analysis. The statistical significance was indicated as asterisks (*). A two-sided *p*-value of <0.05 was considered to be statistically significant (*p < 0.05, **p < 0.01, ***p < 0.001).

## Results

### A20 links glucose metabolism in HCC cell lines

**Fig. 1:**
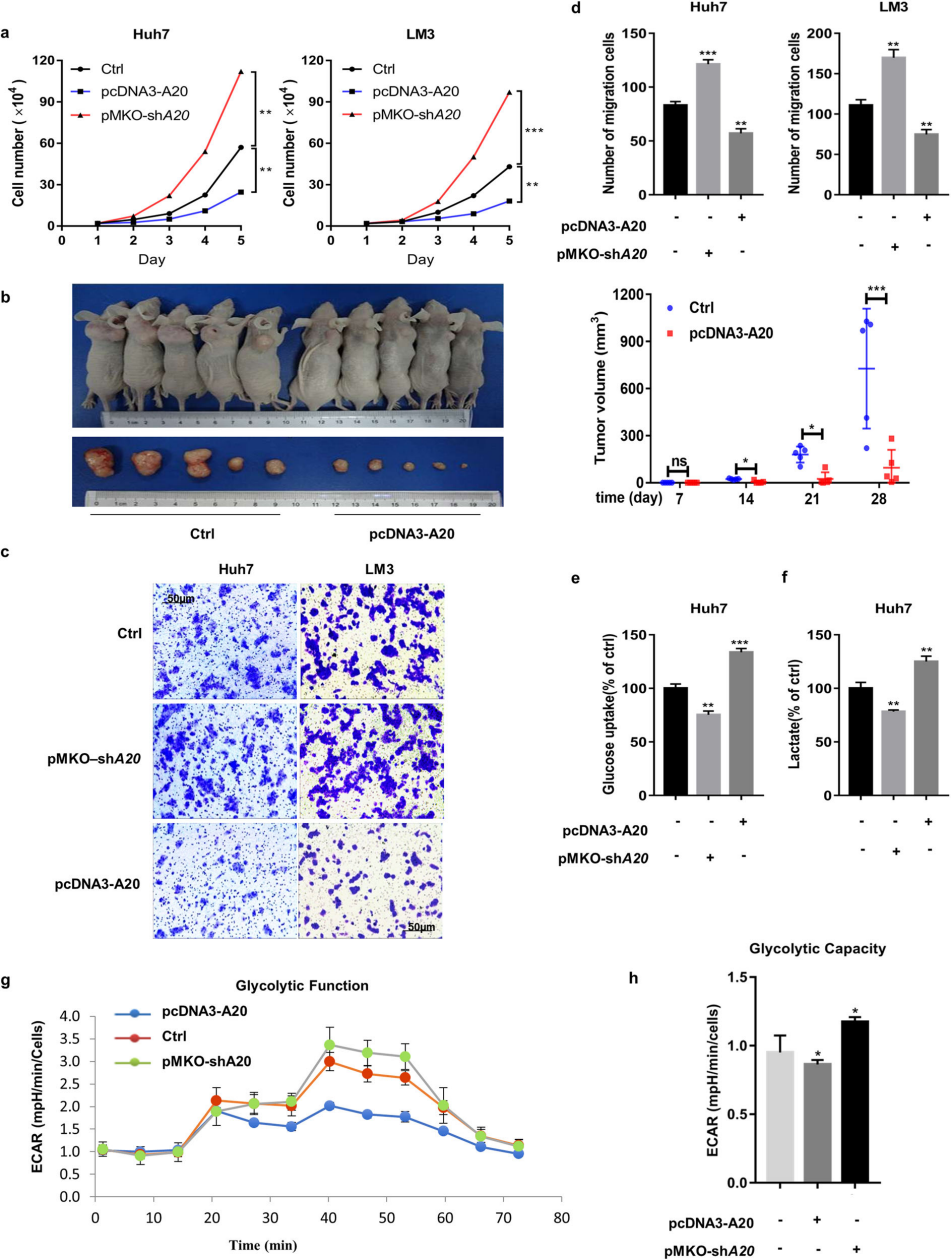
A20 is linked to glucose metabolism in HCC cells. **a** A20 suppresses proliferation in Huh7 and LM3 cells. Cell proliferation was determined by cell counting. The two-tailed Student’s *t*-test was used. **b** A20 decreases HCC tumor growth in vivo. Huh7 cells stably expressing A20 and empty vector counterpart cells were subcutaneously injected into the bilateral flanks of nude mice. At 4 weeks after injection, tumors from 10 mice were extracted and photographed (left panel). Tumor diameters were measured at the indicated time points, and tumor volumes were calculated (right panel). Significant differences were evaluated by *t*-test. **c** A20 suppresses the migration of Huh7 and LM3 cells. Huh7 and LM3 cells were transfected with pcDNA3-A20 or pMKO-sh*A20* plasmids, and cell migration was analyzed by Transwell experiments. **d** Quantitative analysis of cell migration was performed by ImageJ. The numbers of migrated cells (mean ± S.D.) from three independent experiments. **e**, **f** A20 suppresses cell glucose uptake and lactate production. pcDNA3-A20 or pMKO-sh*A20* plasmids was transfected in Huh7 and LM3 cells, respectively, and cellular glucose uptake and lactate production were detected via glucose uptake assay and lactate colorimetric assay, respectively. Error bars represent ± S.D. for triplicate experiments. **g** A20 inhibits cell glycolysis. The real-time assessment of the extracellular acidification rate (ECAR) in cultured cells was examined by Seahorse XFe96 analyzer. **h** Relative glycolytic capacity was normalized to the cell number (means ± S.D., *n* = 3). (The two-tailed Student’s *t*-test was used. The symbol * shows statistically significant differences with *p < 0.05, **p < 0.01, and ***p < 0.001).

Several studies have shown that ectopic expression of A20 is related to cancer progression. To investigate whether A20 is essential for malignant progression of HCC, we established A20 overexpressing or knockdown stable Huh7 and LM3 cell lines and their empty vector counterparts (Supplementary Fig. S1a). A20 knockdown enhanced cell proliferation, clone formation, and migration, while A20 overexpression suppressed these capacities in HCC cells, both in vitro and in vivo (Fig. 1a–d and Supplementary Fig. S1b).

Previous study showed the effect of A20 on NF-κB activity contribute to the impact of A20 on tumor growth^19^. The deregulation of glucose metabolism directly regulates HCC development, and our previous results showed that A20 participated in the progression of HCC. These findings brought our attention to the relationship between A20 and glucose metabolism. To examine whether A20 altered glucose metabolism, we transfected Huh7 cells with A20 overexpressing or knockdown plasmids and measured glucose uptake and lactate production. Both glucose uptake and lactate production were significantly reduced in A20 overexpressing cells compared to the control group, whereas A20 knockdown cells did the opposite (Fig. 1e, f). To corroborate these results, we evaluated glycolytic capacity by measuring the extracellular acidification rate (ECAR) under conditions where cells were supplied sequentially with glucose to feed glycolysis, then the ATP synthase inhibitor oligomycin to drive glycolysis to maximal capacity and then the glucose analog 2-deoxyglucose (2-DG) to block glycolysis. Huh7 cells devoid of A20 displayed substantially increased glycolytic capacity, whereas overexpressing A20 cells showed substantially reduced glycolytic capacity (Fig. 1g, h). These findings demonstrate that A20 plays a negative role in HCC proliferation, migration, and glucose metabolism.

### The metabolic enzyme PFKL is an A20-interacting protein

**Fig. 2:**
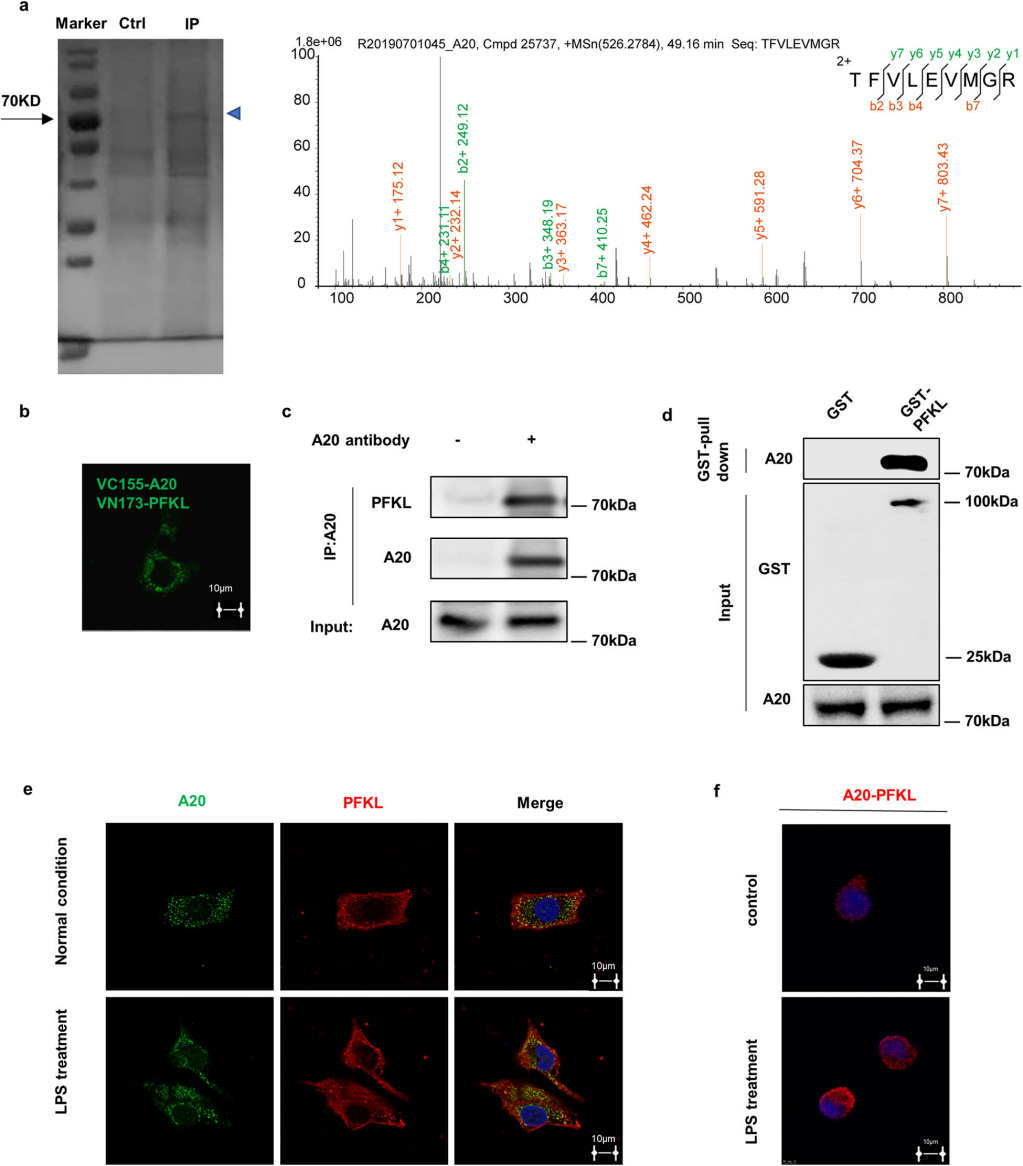
The metabolic enzyme PFKL is an A20-interacting protein. **a** LC-MS/MS identified an interaction between PFKL and A20. **b**, **c** PFKL binds with A20 directly. The interaction between endogenous A20 and PFKL was determined by co-IP and western blotting (**b**). Exogenous VC155-A20 and VN173-PFKL plasmids were transfected into Huh7 cells. Representative confocal pictures of BiFC signals (green) in Huh7 cells are shown (**c**). **d** GST-PFKL can readily pull-down A20. BL21 *E. Coli* were transformed with pGEX-6P-1-GST-PFKL plasmid and induced by isopropyl-b-D-thiogalactoside. Protein was purified by GST antibody-conjugated columns and incubated with Huh7 cell lysates and then repurified through immunoprecipitation and subjected to western blotting. **e**, **f** LPS enhances the interaction between A20 and PFKL. Huh7 cells were cultured with or without LPS for 4 h as indicated and then processed for double immunofluorescence with antibodies against PFKL (green) and A20 (red). Merged images of both channels are shown on the right. Bar: 10 mm (**e**). LPS promotes endogenous PFKL binding with A20 in Huh7 cells. Huh7 cells were pretreated with MG132 for 6 h, then with or without LPS for 4 h as indicated, followed by proximity ligation (Duolink®) assay. Confocal images of the PLA reaction between A20 and PFKL in Huh7 cells. The PLA signal is in red, and DAPI is in blue. Representative data from 3 independent biological experiments (**f**).

To further investigate the mechanism underlying the role of A20 in tumor development and glycolysis suppression, we searched for potential ubiquitination substrates of A20 by screening for potential A20-interacting proteins. We performed IP and LC-MS/MS analysis to identify A20-associated proteins. PFKL was identified as one of the proteins associated with A20 function (Fig. 2a and Supplementary Fig. S2). Several known A20-interacting proteins, including RIP1, TRAF6, and CIAP1, were also among the list of potential A20-interacting proteins identified by the LC-MS/MS assays, validating our approach. Bimolecular Fluorescence Complementation (BiFC) relies on the properties of the N- and C-terminal fragments of fluorescent proteins to fluoresce once they are brought in close proximity. By using this system, we validated the existence of direct interactions between A20 and PFKL (green fluorescence), which were fused to a non-fluorescent N- or C-terminal fragment, respectively (Fig. 2b).

The interaction of endogenous PFKL with A20 in Huh7 cells was confirmed by using Co-IP followed by western blotting assay in Huh7 cells (Fig. 2c). Further, we performed an in vitro GST pull-down assay to identify whether PFKL interacts with A20 directly. GST-PFKL protein was purified from *E. coli*, and the results showed that GST-PFKL interacted with A20 (Fig. 2d). Confocal microscopy demonstrated A20 localization in the cytoplasm and colocalization of A20 with PFKL in HCC cells. As a negative immune regulator, A20 can be expressed in large quantities after being stimulated by LPS, TNF, CD40, and other cytokines to exert ubiquitin ligase activity. Here, we treated Huh7 cells with lipopolysaccharide (LPS). The results showed that the interaction between A20 and PFKL was enhanced, in contrast with the control (Fig. 2e). To further confirm the A20-PFKL interaction, in-cell experiments were performed using the Duolink proximity ligation assay (PLA). Briefly, cells were fixed and incubated with anti-A20 and PFKL antibodies, followed by the addition of plus and minus PLA probes with attached short DNA strands, followed by visualization with confocal microscopy. The results showed that the in-cell A20-PFKL complex (red fluorescence) was more abundant under LPS stimulation, compared with the control group (Fig. 2f). This finding is consistent with the confocal microscopy data, which confirmed the direct interaction between A20 and PFKL in HCC cells. Taken together, these results indicate that PFKL physically interacts with A20 directly.

### A20 downregulates PFKL protein levels at the post-transcriptional level

**Fig. 3:**
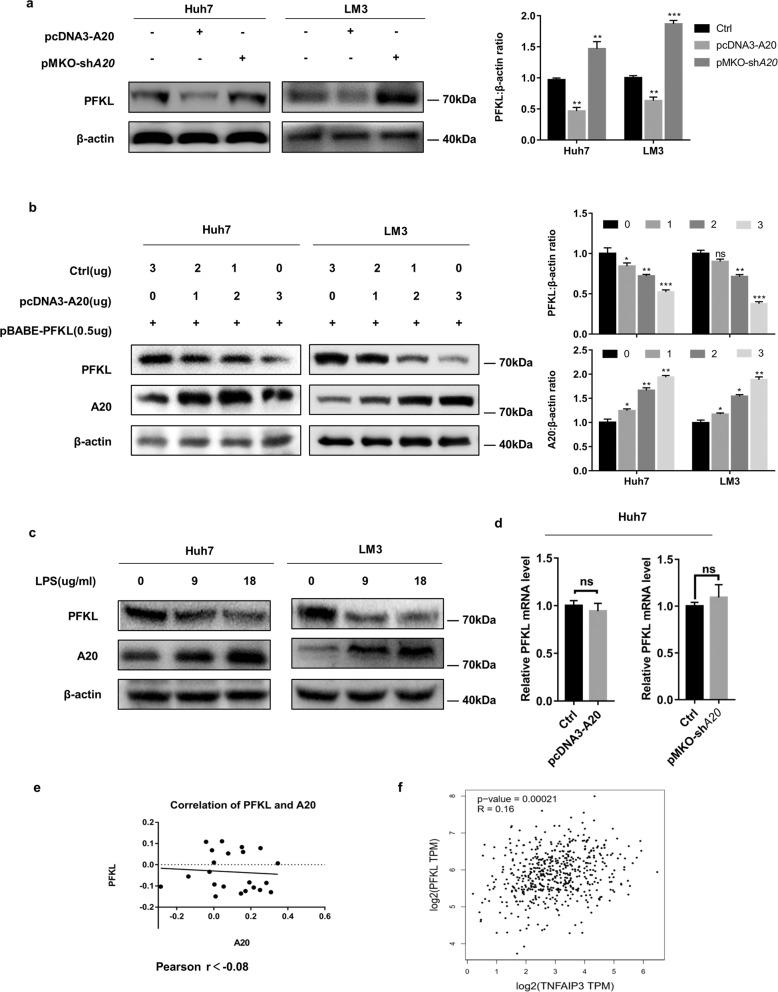
A20 downregulates PFKL protein levels by post-transcriptional modification. **a** A20 decreases the PFKL protein level. Huh7 and LM3 cells were transfected with pcDNA3-A20 or pMKO-sh*A20* plasmids. The protein levels of PFKL were determined by standard western blotting (left panel). The relative PFKL protein compared with the β-actin level was quantified (right panel). **b** A20 reduces PFKL protein level in a dose-dependent manner. Huh7 cells were transfected with the PFKL vector together with varying amounts of A20 or empty control (Con) vectors, and the protein levels of PFKL were determined by standard western blotting (left panel). The relative PFKL protein compared with the β-actin level and relative A20 protein compared with the β-actin level was quantified (right panel). **c** LPS treatment increases the endogenous A20 protein level and decreases the PFKL protein level. Huh7 cells were treated with 9 and 18 μg/mL LPS for 4 h. Untreated cells were used as controls. The expression level of A20 protein was determined by western blotting. **d**–**f** A20 decreases PFKL expression at the post-transcriptional level. PFKL mRNA was determined by qPCR and normalized against β-actin. Error bars represent ± S.D. of triplicate experiments. The two-tailed Student’s *t*-test was used. NS denotes no significance (**d**). Scatterplots revealed that A20 and PFKL were not correlated at the mRNA level. Clinical data of non-cancerous liver samples and hepatocellular carcinoma samples were based on GSE364 (**e**). Data from the GEPIA website showed that A20 has no correlation with PFKL at the mRNA level (*r* = 0.16) (**f**). (The two-tailed Student’s *t*-test was used. The symbol * shows statistically significant differences with *p < 0.05, **p < 0.01, and ***p < 0.001).

Ubiquitin ligases are important regulators of intracellular protein stability, protein subcellular localization, structure, and function. To investigate whether A20 regulates PFKL protein stability in HCC cells, we transferred pcDNA3-A20 or pMKO-sh*A20* plasmids into Huh7 and LM3 cells. As is shown in Fig. 3a, A20 overexpression downregulated the PFKL protein level, while knockdown of A20 did the opposite. To further confirm this phenomenon, Huh7 and LM3 cell lines were co-transduced with vectors expressing A20 and PFKL, respectively. Ectopic A20 expression reduced PFKL protein levels in both cell lines in a dose-dependent manner (Fig. 3b). In addition, we treated Huh7 and LM3 cells with LPS at 0, 9 and 18 ng and found a dose-dependent increase in A20 protein expression, but a significant decrease in the PFKL protein level (Fig. 3c).

Previous studies report that ubiquitin ligase modulates the degradation of multiple proteins^28^. To investigate whether A20 downregulates PFKL protein by ubiquitination, we first transfected A20 overexpressing or knockdown plasmids into HCC cells and examined PFKL mRNA levels in the control group. Neither ectopic A20 expression nor knockdown of endogenous A20 affected PFKL mRNA levels as analyzed by quantitative Real-Time PCR (Fig. 3d). Furthermore, we performed datamining of HCC data sets from the GEO Datasets using Pearson’s correlation analysis. The result showed that A20 expression was significantly uncorrelated with PFKL at the mRNA level (*r* < −0.08) (Fig. 3e). Consistent with these results, data from the GEPIA website also showed that A20 has no correlation with PFKL at the mRNA level (*r* = 0.16) (Fig. 3f). Collectively, these results indicate that A20 negatively regulates PFKL at the protein level in HCC.

### A20 promotes ubiquitination and degradation of PFKL

**Fig. 4:**
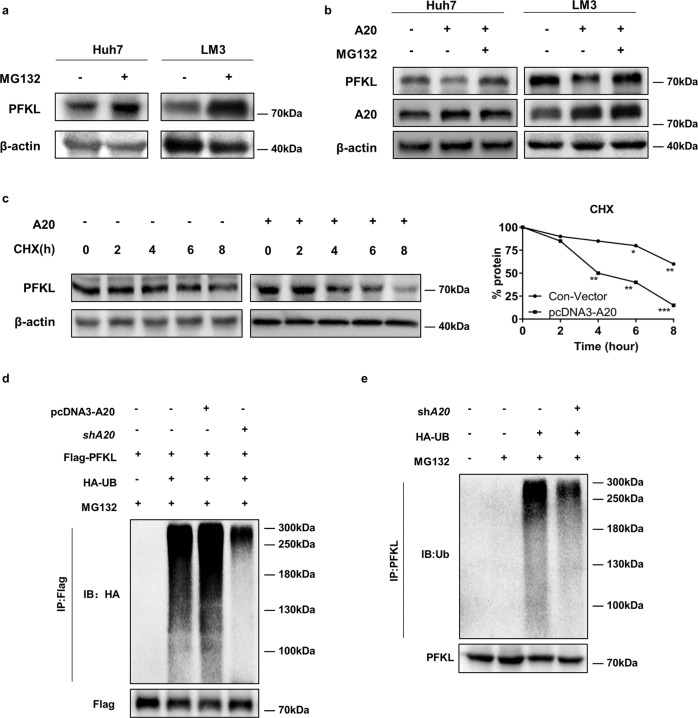
A20 promotes ubiquitination and degradation of PFKL. **a** MG132 treatment led to the accumulation of PFKL protein levels. Huh7 and LM3 cells were treated with MG132. Cell lysates were directly subjected to western blotting. **b** MG132 rescues PFKL protein reduction induced by A20 overexpression. Huh7 and LM3 cells were transfected with pcDNA3-A20 plasmid, followed by MG132 treatment. The protein expression level of PFKL and A20 was determined by western blotting. **c** A20 overexpression shortens the half-life of PFKL. Huh7 cells were transfected with pcDNA3-A20 or vector plasmids. A CHX chase experiment was performed and PFKL protein was determined by western blotting (left panel). The right panel showcases the relative protein amounts of different groups. Error bars represent ± S.D. of triplicate experiments. The two-tailed Student’s *t*-test was used. *p < 0.05, **p < 0.01, and ***p < 0.001. **d** A20 promotes PFKL degradation through ubiquitination. Huh7 cells were transfected with the indicated plasmids. After MG132 treatment, the ubiquitination level of purified flag-PFKL protein was determined. **e** A20 knockdown decreases the ubiquitination level of endogenous PFKL. Huh7 cells were transfected with the indicated plasmids. After MG132 treatment for 6 h, cells were lysed for co-IP assay. Endogenous PFKL was immunoprecipitated by anti- PFKL antibody and probed for total ubiquitin.

Numerous cellular proteins are degraded via the proteasomal pathway. To address whether A20 is a potential PFKL E3 ligase, we next investigated whether A20 promoted PFKL degradation via UPS. We treated Huh7 and LM3 cells with the proteasome inhibitor MG132 and determined the protein level of PFKL by western blotting. As shown in Fig. 4a, cells treated with MG132 had a significant increase in the PFKL protein. Moreover, we found that MG132 effectively abrogated the A20-mediated degradation of PFKL, which confirmed that PFKL was degraded via the proteasomal pathway (Fig. 4b). A cycloheximide (CHX) chase experiment indicated that PFKL is an unstable protein in cells grown with A20 overexpression, with a half-life of approximately 6 h (Fig. 4c).

To investigate whether A20 promoted PFKL degradation through ubiquitination, we then performed in vivo ubiquitination assays. Huh7 cells were transfected with Flag-PFKL and HA-ubiquitin (HA-Ub) plasmids, followed by A20 overexpression or silencing. The results revealed that A20 overexpression increased the ubiquitination of PFKL, while the knockdown of endogenous A20 effectively decreased PFKL ubiquitination (Fig. 4d and Supplementary Fig. S3a). On this basis, we explored the impact of A20 expression level on endogenous PFKL ubiquitination. As shown in Fig. 4e, knockdown of A20 effectively decreased endogenous PFKL ubiquitination. From these results, we conclude that A20 markedly promotes the ubiquitination and degradation of PFKL in HCC cells.

### A20 inhibits cell proliferation and migration by downregulating glucose metabolism

**Fig. 5:**
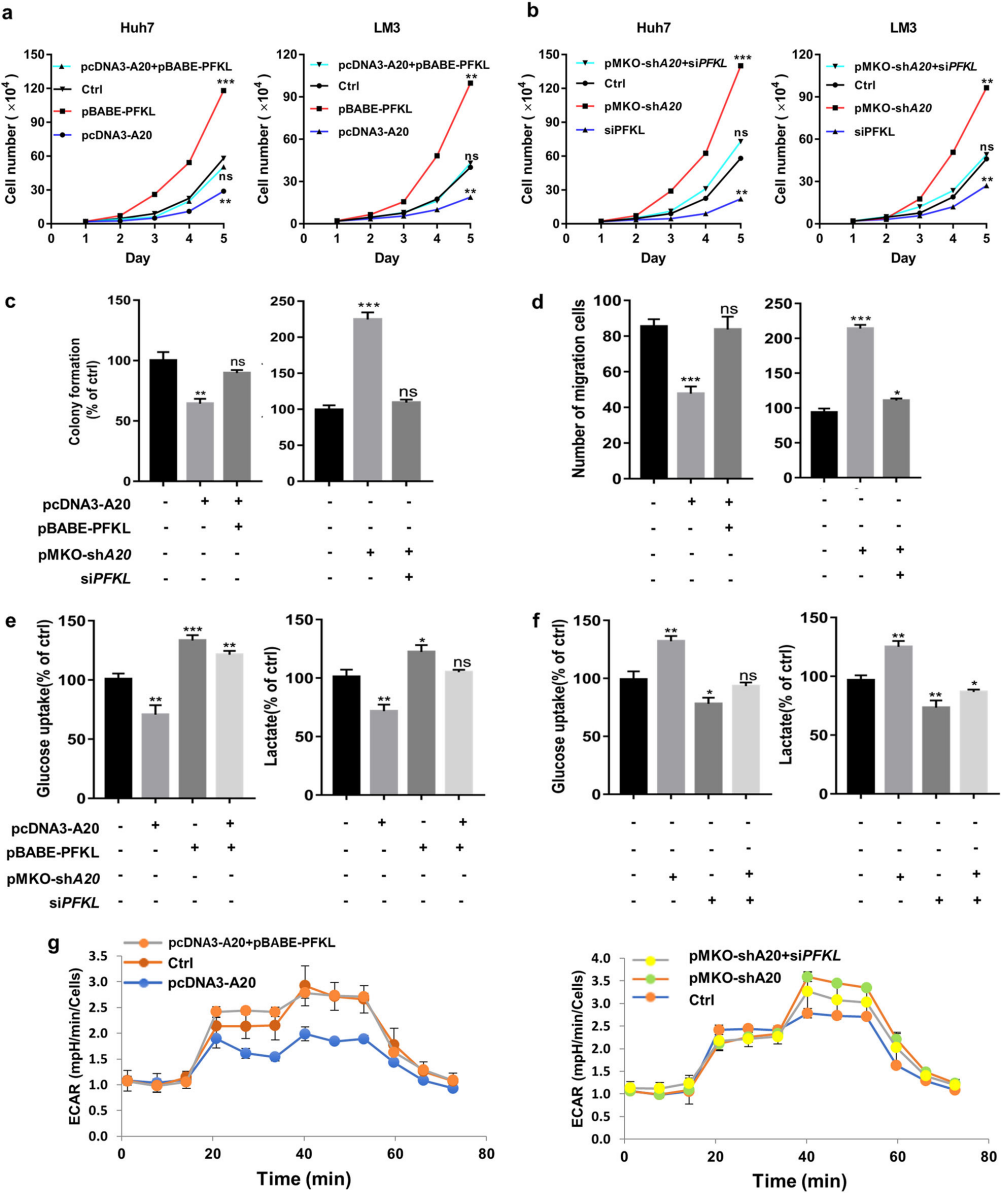
A20 inhibits cell proliferation and migration by downregulating glucose metabolism. **a**, **b** A20 inhibits HCC cell proliferation through PFKL. Huh7 and LM3 cells were transfected with the indicated plasmids respectively. Proliferative ability was determined by cell counting assay at the indicated times. Data represent the means ± S.D. of three independent experiments. **c** A20 inhibits HCC cell colony formation through PFKL. The indicated plasmids were co-transfected into Huh7 cells. Proliferative ability was determined by colony formation experiment at 10 days in culture. Error bars represent ± S.D. for triplicate experiments. **d** A20 inhibits HCC cell migration through PFKL. The indicated plasmids were co-transfected into Huh7 cells. Cell migration capacity was analyzed by Transwell experiments. The numbers of migrated cells (mean ± S.D.) from three independent experiments. **e**, **f** A20 inhibits HCC cell glucose consumption and lactate excretion by downregulating PFKL. The indicated plasmids were co-transfected into Huh7 cells. Cell glucose consumption and lactate excretion were detected by glucose uptake assay and lactate colorimetric assay, respectively. Error bars represent ± S.D. of triplicate experiments. **g** A20 inhibits HCC cell glycolysis through PFKL. ECAR was examined using a Seahorse XFe96 analyzer. (The two-tailed Student’s *t*-test was used. The symbol * shows statistically significant differences with *p < 0.05, **p < 0.01 and ***p < 0.001).

Aerobic glycolysis promotes tumor cell proliferation and migration. The conversion of fructose-6-phosphate (F6P) to fructose-1,6- bisphosphate (F1,6BP) by 6-phosphofructokinase (PFK) is the first committed step of glycolysis and is essentially irreversible. Considering the critical role of PFK1 in cancer glucose metabolism, we next verified whether PFKL is involved in A20-mediated regulation of HCC progression. Rescue experiments in Huh7 and LM3 cells were performed. We ectopically overexpressed PFKL in A20 overexpressing Huh7 and LM3 cell lines and found that PFKL overexpression effectively abrogated the repression of proliferation mediated by A20 (Fig. 5a). Conversely, the increased cell proliferation induced by A20 depletion was specifically blocked by the knockdown of *PFKL* in both the Huh7 and LM3 cell lines (Fig. 5b and Supplementary Fig. S3b). Accordingly, a colony formation assay showed similar trends in Huh7 cells (Fig. 5c and Supplementary Fig. S3c). As we previously described, A20 knockdown promoted the migration of HCC cells. However, the knockdown of PFKL significantly repressed migration capacity in Huh7 cells with A20 knockdown. Similarly, PFKL overexpression significantly blocked the reduced cell migration caused by A20 overexpression in Huh7 cells (Fig. 5d and Supplementary Fig. S3d). Taken together, these findings suggest that A20-induced suppression of HCC proliferation and migration is mainly mediated through inhibition of PFKL expression.

To verify that the downregulation of PFKL induced by A20 is responsible for the decreased glucose consumption of HCC cells, we ectopically overexpressed PFKL and found an increase in glucose uptake and lactate production in A20 overexpressing Huh7 cells. In contrast, specific knockdown of PFKL by siRNA clearly blocked the increased glucose uptake and lactate production in A20 knockdown Huh7 cells (Fig. 5e, f). To confirm these results, we used a Seahorse metabolic analyzer to measure the extracellular acidification rate (ECAR) in Huh7 cells. The results showed that overexpression of A20 can make markedly reduce glycolytic function compared to control cells, whereas it can be restored by re-expressing PFKL. Consistent with previous results, loss of A20 markedly increased glycolytic function compared to control cells and was blocked by PFKL-siRNA (Fig. 5g and Supplementary Fig. S3e). Collectively, our data demonstrate that A20 inhibits glycolysis through downregulation of PFKL, thereby suppressing HCC cell proliferation and migration.

### A20 expression is inversely correlated with PFKL in HCC patients

**Fig. 6:**
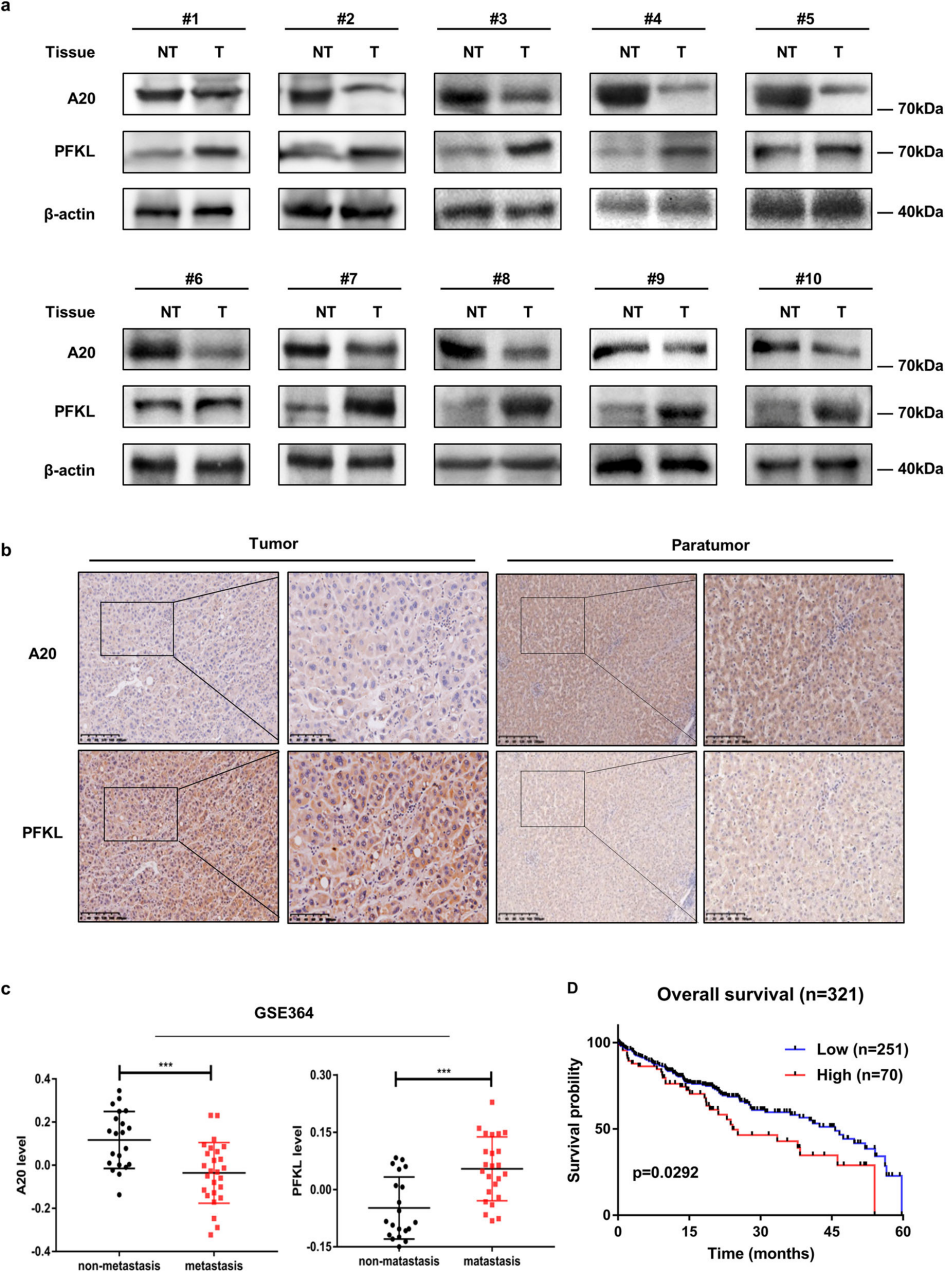
A20 expression is inversely correlated with PFKL in HCC patients. **a** A20 expression is inversely with PFKL. Human HCC samples were paired as tumor tissue (designated as T) and adjacent non-tumor tissue (designated as NT). Samples were lysed and directly subjected to western blotting. 10 pairs of samples showcasing an inverse correlation are shown. **b** Immunohistochemical staining of A20 and PFKL proteins in tumor and adjacent non-tumor tissues. A20 expression was negatively correlated with the PFKL level in human HCC specimens (magnification, left panel, ×100; right panel, ×400). **c** A20 and PFKL mRNA levels are inversely correlated in non-metastatic tissues and metastatic tissues. Scatterplot data are from clinical data sets from GSE364. **d** Kaplan–Meier overall survival curves of HCC patients in the Protein Atlas website showing that high expression of PFKL significantly correlates with poor overall survival.

The finding that the A20-PFKL axis regulates HCC cell migration and proliferation prompted us to examine both A20 and PFKL protein levels in human HCC samples. We collected 10 matched HCC samples and adjacent non-cancerous tissues and examined A20 and PFKL protein levels by western blotting. The levels of PFKL were shown to be significantly higher in HCC tissues compared to adjacent non-cancerous samples, while the levels of A20 were lower in HCC samples compared to adjacent non-cancerous tissues (Fig. 6a). To substantiate the finding that A20 promotes ubiquitination and degradation of PFKL, we further verified the level of both A20 and PFKL by immunohistochemistry in paraffin-embedded tissues. In most samples, A20 expression was inversely correlated with PFKL protein levels in cancerous tissues and adjacent non-cancerous tissues (Fig. 6b). Statistical analysis of quantified images indicated that the differences between tumor and paratumor tissues in A20 protein levels (*P* < 0.001) and in PFKL protein levels (*P* < 0.001) were all significant (Supplementary Fig. S4a). Consistent with Fig. 1c, data from the GEO Datasets showed that the A20 mRNA level was higher in non-metastatic tissues than metastatic tissues, while the PFKL mRNA level was higher in metastatic tissues than in non-metastatic tissues (Fig. 6c). Importantly, data from the Protein Atlas website showed that patients with higher PFKL expression had poor overall survival (Fig. 6d). In addition, data from the GEPIA website showed that patients with higher PFKL expression had poor disease-free survival (Supplementary Fig. S4b). Taken together, these results strongly indicate that high expression of PFKL is significantly correlated with downregulation of A20 and predicts lower survival in HCC patients.

## Discussion

Aerobic glycolysis is the most prominent metabolic feature associated with malignant transformation^29^. Cancer cells display a dramatic increase in glucose uptake, for the high demand of metabolic intermediates and biosynthesis to support rapid cell growth^30^. Imbalanced glucose metabolism plays an important role in HCC progression. Here, we show that A20 inhibits glycolysis by promoting PFKL degradation through UPS. Additionally, A20 knockdown promotes HCC cell proliferation and migration. These findings suggest that A20 is an essential regulator of HCC glucose metabolism and progression.

Altered A20 expression implies its role in cancer pathogenesis. The expression of A20 is constitutive in lymphoid tissues and inducible in various cells, such as endothelial cells, islet cells, and a variety of tumor cells^31^. The expression profile of A20 in cancers is cell-type-dependent. High expression of A20 was found in some solid cancers such as bladder cancer, nasopharyngeal carcinoma, and squamous cell carcinoma^14,32,33^. On the other hand, downregulation of A20 was discovered in lymphomas and some solid cancers such as pancreatic cancer and colorectal tumor, and may be involved in breast cancer and brain metastasis^10,34^. Previous studies show that A20 expression is absent in normal liver, but is present in hepatitis tissue^35,36^. A20 is also expressed in HCC tissues probably due to the inflammatory environment of HCC. Consistently, our results showed that A20 was aberrant expressed in HCC. A20 expression was elevated in adjacent non-tumor tissues compared to HCC tissues

Currently, the mechanism underlying A20’s function in tumor suppression is not well-defined. H. Chen et al. observed that increased expression of A20 was negatively correlated with poor prognostic factors in patients with HCC. Patients with higher A20 expression had better disease-free survival and overall survival time than those with lower A20 expression^37^. Recent study reported that knockdown of A20 can alter the tumor metabolism. Here, we identified the glycolysis rate-limiting enzyme PFKL as a substrate of A20 involved in HCC progression. Interestingly, a higher PFKL expression level is correlated with significantly lower overall survival and disease-free survival (https://www.proteinatlas.org/ and http://gepia.cancer-pku.cn/). In rapidly proliferating cancer cells, a high expression level of PFKL serves as a switch for glycolysis. Our study revealed that overexpression of A20 promotes PFKL degradation through UPS, which leads to decreased glycolysis and subsequently inhibits HCC cell proliferation and metastasis. Therefore, A20 and PFKL protein expression status could be attractive candidate markers to stratify patients with different stage HCC into distinct subgroups and help guide individualized treatment. However, the potential ubiquitination site in PFKL has not yet been identified. Thus, further proteomic analyses need to be performed to identify the modified lysine residue. We propose that ubiquitination of certain lysine residues can cause conformational changes in PFKL, making PFKL accessible for recognition by the proteasome and inhibiting its enzyme activity.

**Fig. 7:**
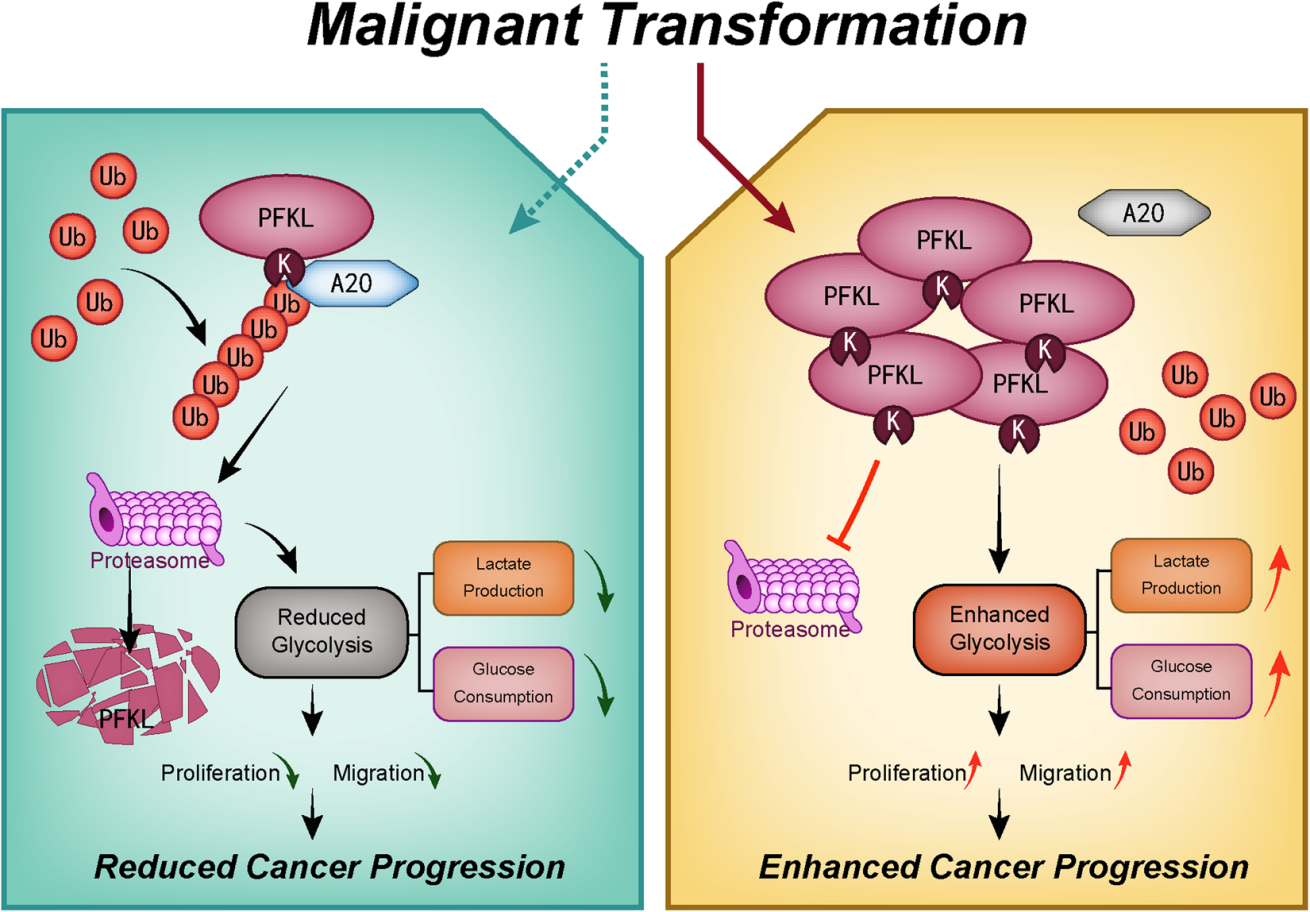
Working model. A20 upregulation promotes PFKL degradation through UPS. Malignant transformation of HCC results in decreased expression of A20 and subsequently reduced PFKL degradation. Accumulation of PFKL facilitates rapid cell proliferation and migration, therefore promoting HCC growth.

Over the past decade, to inhibit tumor development, many efforts have been made to therapeutically target glucose metabolism^38,39^. Our study demonstrates that A20 targets PFKL for ubiquitination-mediated degradation, while the regulation signals remain unknown. Hence, we will next explore upstream effectors that mediate A20 function to suppress glucose metabolism and HCC progression. In conclusion, these data suggest that the E3 ligase A20 acts as a tumor suppressor to promote PFKL degradation through UPS, thus suppressing the Warburg effect and inhibiting HCC cell proliferation and metastasis (Fig. 7). A better understand of A20-mediated PFKL ubiquitination may, therefore, lead to the development of combination therapies and become a potential strategy for future anti-HCC therapy.

## Supplementary information

Supplementary Figure Legends

Supplementary Figure S1

Supplementary Figure S2

Supplementary Figure S3

Supplementary Figure S4
