# Supplementary material for: A20 targets PFKL and glycolysis to inhibit the progression of hepatocellular carcinoma
Source: Cell Death Dis. 2020 Feb 3;11(2):89. doi: 10.1038/s41419-020-2278-6 (PMC6997366; doi:10.1038/s41419-020-2278-6)
Supplement: Supplementary file 1 — Supplementary Figure Legends [file 41419_2020_2278_MOESM1_ESM.docx]

## SUPPLEMENTAL INFORMATION

**A20 targets PFKL and glycolysis to inhibit the progression of hepatocellular carcinoma**

**Yilu Feng^1#^, Ye Zhang^1#*^,** Yi Cai^2^, Ruijie Liu^3^, Miaolong Lu^1^, Tangzhiming Li^4^, Ying Fu^1^, Ming Guo^1^, Huichao Huang^1^, Yifu Ou^5^, Yongheng Chen^1*^

**Inventory of Supplemental Information**

**Supplementary Fig. S1: Linked to Figure 1**

**Supplementary Fig. S2: Linked to Figure 2**

**Supplementary Fig. S3: Linked to Figure 4 and 5**

**Supplementary Fig. S4: Linked to Figure 6**

**Supplementary Fig. S1. A20 is linked to glucose metabolism in HCC cells. (a)**Verification of Huh7 and LM3 stable cell lines. Knockdown efficiency and overexpression levels of A20 in Huh7 and LM3 cells were determined by western blotting and normalized against β-actin. **(b)** A20 inhibits cell colony formation capacity in HCC cells. Huh7 and LM3 stable cells were cultured for 14 days, and analyzed by a colony formation assay. The symbol * showeds statistically significant differences with *p < 0.05, **p< 0.01 and ***p < 0.001. Error bars represent ± S.D. for triplicate experiments.

**Supplementary Fig. S2. The metabolic enzyme PFKL is an A20-interacting protein.** LC-MS/MS identifies glycolytic enzyme, PFKL as a candidate of A20 interacting protein.

**Supplementary Fig. S3. A20 inhibits cell proliferation and migration by downregulating glucose metabolism. (a)** PFKL transfection identification. Huh7 cells transfected with pBABE-PFKL, Cell lysates were directly subjected to western blotting and probed with specific antibodies to flag and β-actin. **(b)** si*PFKL* transfection identification. Huh7 cells were transfected with si*PFKL*, Cell lysates were directly subjected to western blotting and probed with specific antibodies to PFKL and β-actin. **(c)** A20 inhibits HCC cell cloning formation through PFKL. The indicated plasmids were co-transfected into Huh7 cells. Proliferation ability was determined by cloning formation experiment at 10 days cultural. **(d)** A20 inhibits HCC cells migration through PFKL. The indicated plasmids were co-transfected into Huh7 cells. And cells migration capacity was analyzed by transwell experiments. **(e)** A20 inhibits HCC cells glycolysis through PFKL. Relative glycolytic capacity was normalized to the cell number (means ± S.D., n = 3), *p < 0.05, **p< 0.01 and ***p < 0.001 based on the Student’s t test.

**Supplementary Fig. S4. A20 expression is inversely correlated with PFKL in HCC patients. (a)** A total of 10 HCC tissues and 10 paratumor tissues are analyzed. The mean value of multiple samples and standard deviation were presented. **(b)** High expression of PFKL significantly correlates with disease-free survival. Kaplan-Meier disease-free survival curves of HCC patients according to the designated gene expression levels above or below the median value based on the analysis with the GEPIA bioinformatic tool of genomic database.
