# Supplementary figures and images for: A20 targets PFKL and glycolysis to inhibit the progression of hepatocellular carcinoma
Source: Cell Death Dis. 2020 Feb 3;11(2):89. doi: 10.1038/s41419-020-2278-6 (PMC6997366; doi:10.1038/s41419-020-2278-6)

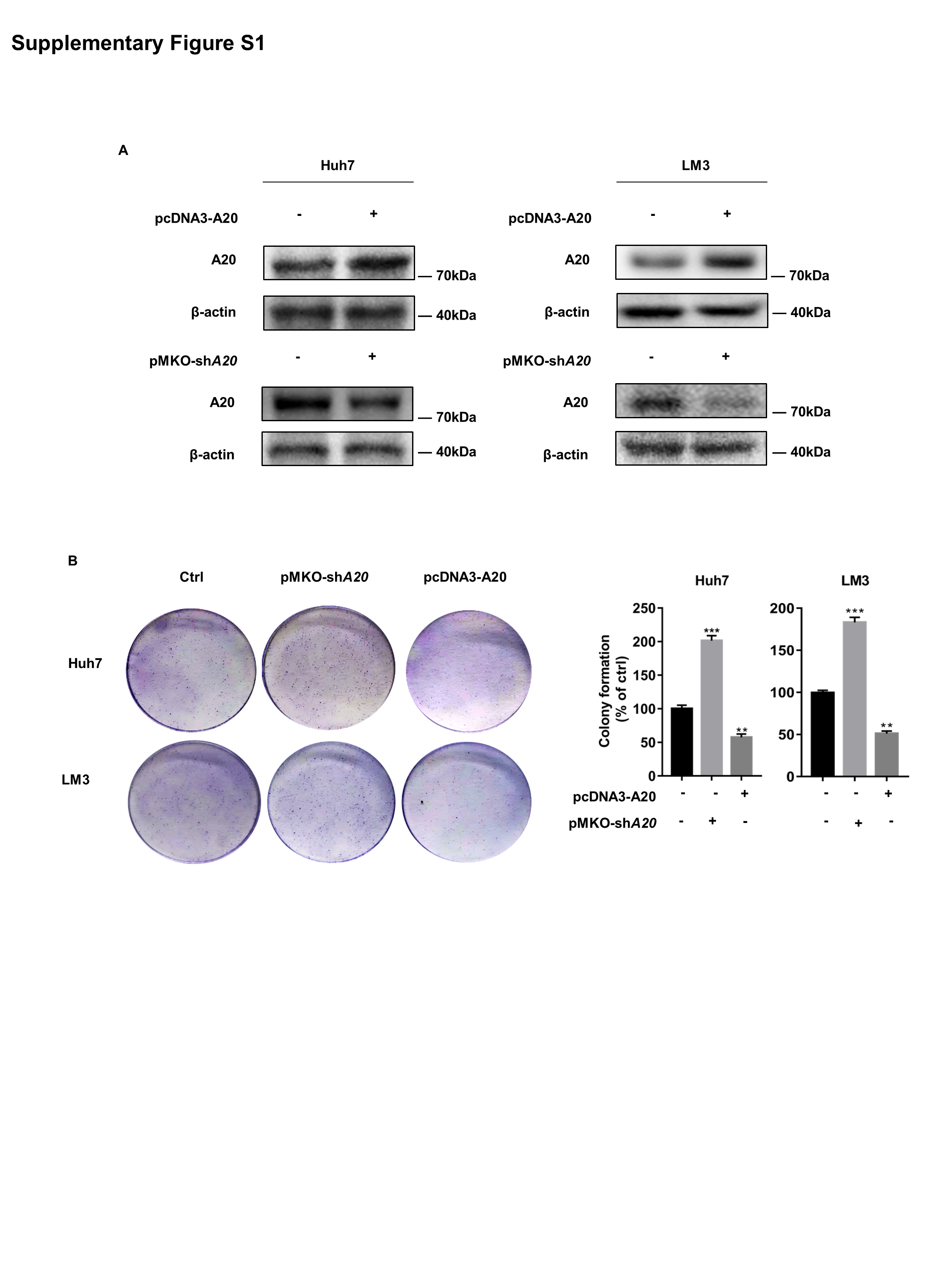

Supplement: Supplementary file 2 — Supplementary Figure S1 [file 41419_2020_2278_MOESM2_ESM.tif]

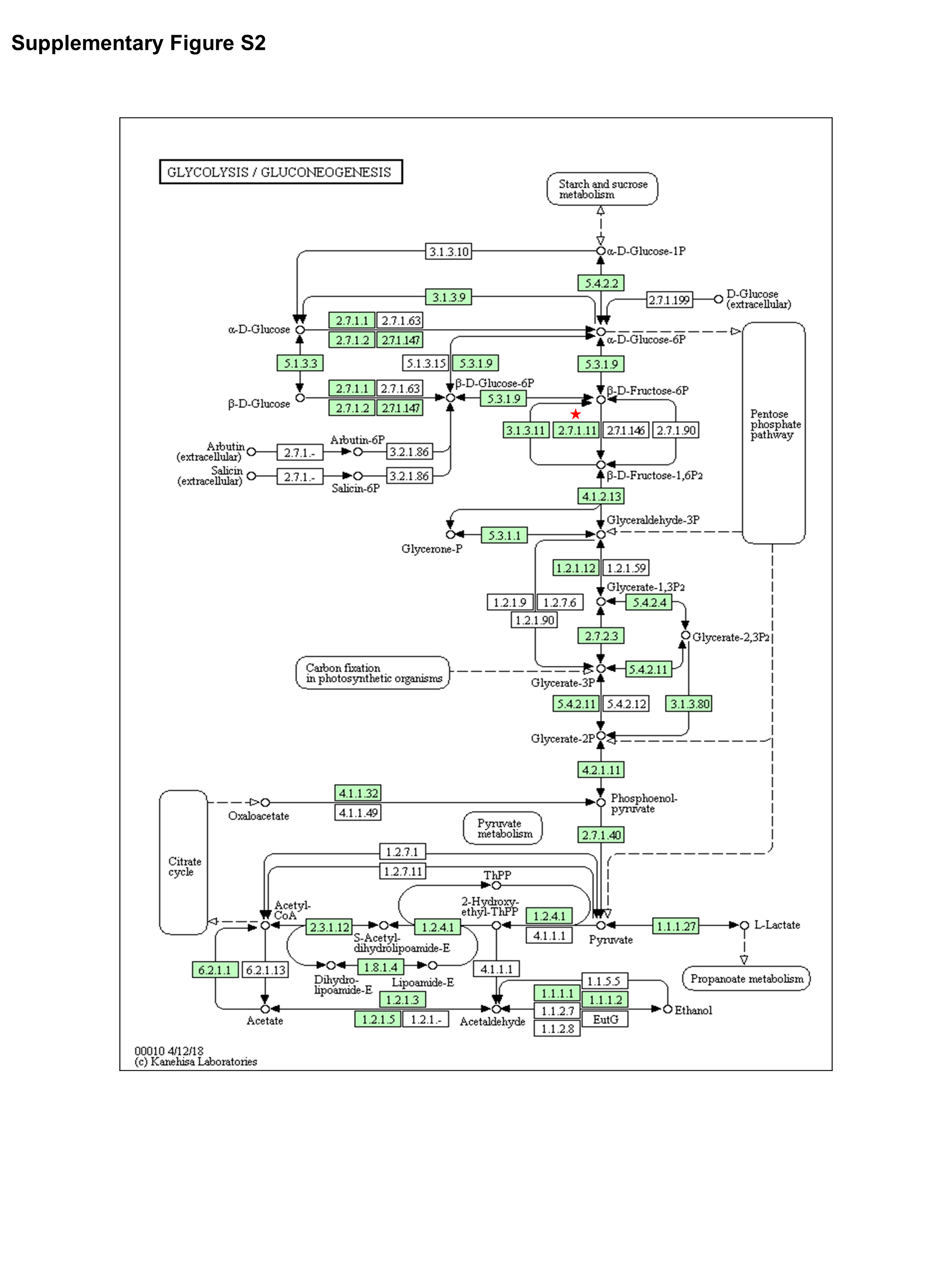

Supplement: Supplementary file 3 — Supplementary Figure S2 [file 41419_2020_2278_MOESM3_ESM.tif]

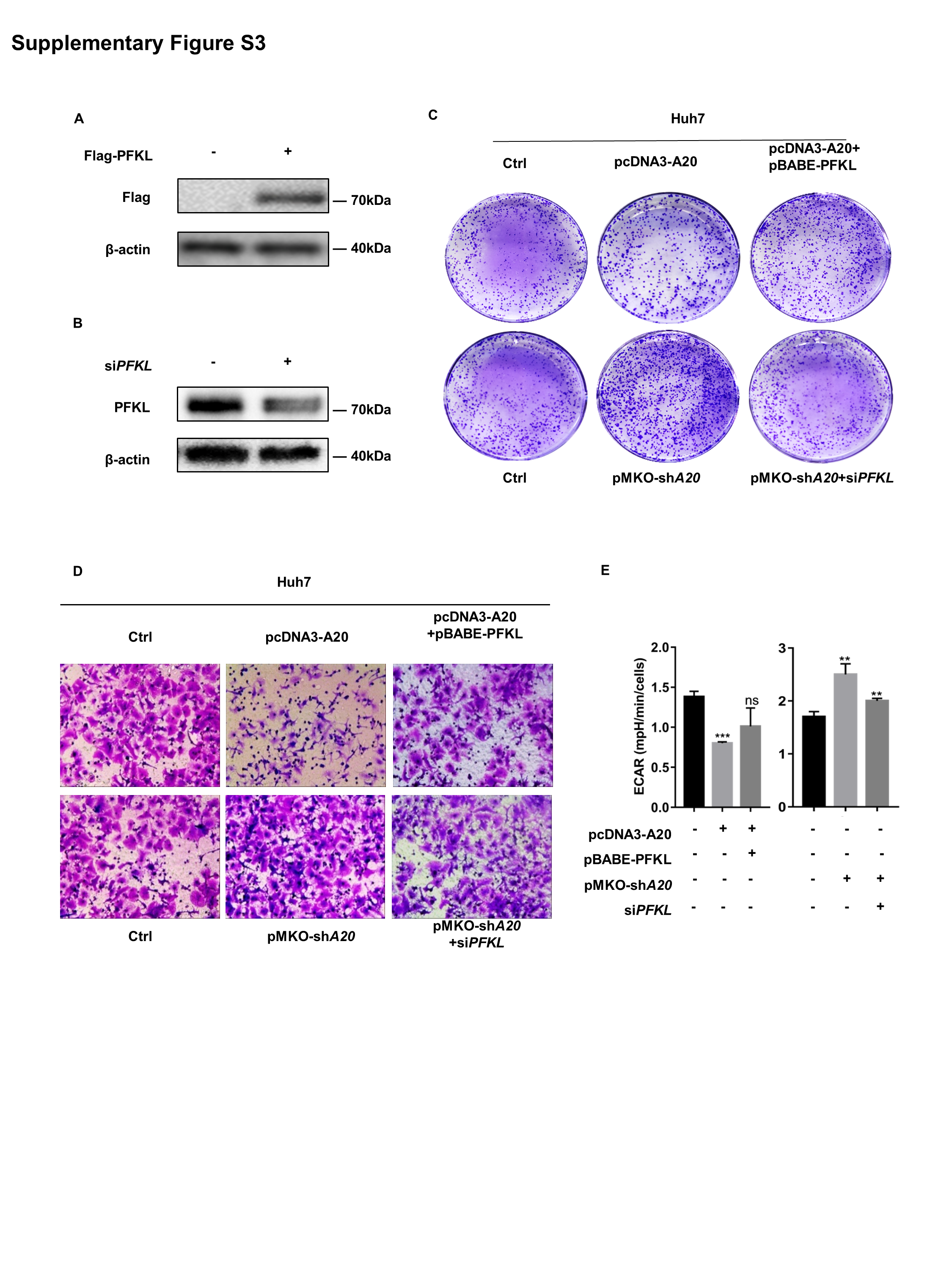

Supplement: Supplementary file 4 — Supplementary Figure S3 [file 41419_2020_2278_MOESM4_ESM.tif]

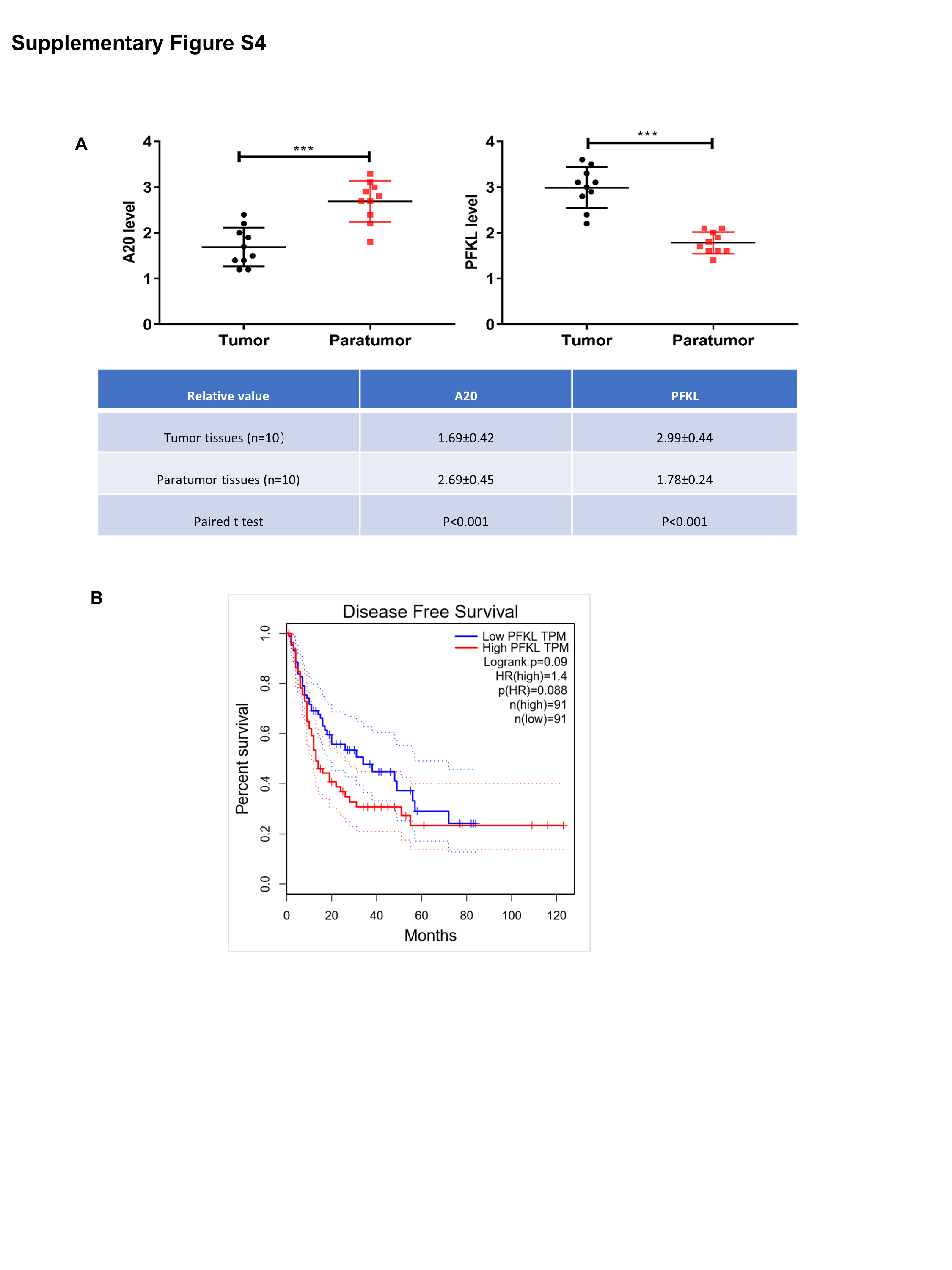

Supplement: Supplementary file 5 — Supplementary Figure S4 [file 41419_2020_2278_MOESM5_ESM.tif]
